# Classifications for Cesarean Section: A Systematic Review

**DOI:** 10.1371/journal.pone.0014566

**Published:** 2011-01-20

**Authors:** Maria Regina Torloni, Ana Pilar Betran, Joao Paulo Souza, Mariana Widmer, Tomas Allen, Metin Gulmezoglu, Mario Merialdi

**Affiliations:** 1 Department of Obstetrics, Sao Paulo Federal University and Brazilian Cochrane Centre, Sao Paulo, Brazil; 2 Department of Reproductive Health and Research, World Health Organization, Geneva, Switzerland; 3 Department of Knowledge Management and Sharing, World Health Organization, Geneva, Switzerland; Institute for Clinical Effectiveness and Health Policy (IECS), Argentina

## Abstract

**Background:**

Rising cesarean section (CS) rates are a major public health concern and cause worldwide debates. To propose and implement effective measures to reduce or increase CS rates where necessary requires an appropriate classification. Despite several existing CS classifications, there has not yet been a systematic review of these. This study aimed to 1) identify the main CS classifications used worldwide, 2) analyze advantages and deficiencies of each system.

**Methods and Findings:**

Three electronic databases were searched for classifications published 1968–2008. Two reviewers independently assessed classifications using a form created based on items rated as important by international experts. Seven domains (ease, clarity, mutually exclusive categories, totally inclusive classification, prospective identification of categories, reproducibility, implementability) were assessed and graded. Classifications were tested in 12 hypothetical clinical case-scenarios. From a total of 2948 citations, 60 were selected for full-text evaluation and 27 classifications identified. Indications classifications present important limitations and their overall score ranged from 2–9 (maximum grade = 14). Degree of urgency classifications also had several drawbacks (overall scores 6–9). Woman-based classifications performed best (scores 5–14). Other types of classifications require data not routinely collected and may not be relevant in all settings (scores 3–8).

**Conclusions:**

This review and critical appraisal of CS classifications is a methodologically sound contribution to establish the basis for the appropriate monitoring and rational use of CS. Results suggest that women-based classifications in general, and Robson's classification, in particular, would be in the best position to fulfill current international and local needs and that efforts to develop an internationally applicable CS classification would be most appropriately placed in building upon this classification. The use of a single CS classification will facilitate auditing, analyzing and comparing CS rates across different settings and help to create and implement effective strategies specifically targeted to optimize CS rates where necessary.

## Introduction

The worldwide rise in cesarean section (CS) rates is becoming a major public health concern and cause of considerable debate due to potential maternal and perinatal risks, cost issues and inequity in access.[1]–[4] The increase in CS rates observed in many developed and middle-income countries contrasts sharply with the very low rates in numerous low-resource settings, along with lack of access to emergency obstetric care. According to recent data, in Middle Africa, only 1.8% of all live birth deliveries occur by CS, compared to 24.3% in North America and 31% and in Central America.[5] The main determinants of this disparity and specific reasons for the increase in CS rates in most of the world remain unclear.

In order to propose and implement effective measures to reduce or increase CS rates where necessary, it is first essential to identify what groups of women are undergoing CS and investigate the underlying reasons for trends in different settings. This requires the use of a classification system that can best monitor and compare CS rates in a standardized, reliable, consistent and action-oriented manner. Such a classification system should be applicable internationally and useful for clinicians and public health authorities. Ideally, such a system should be simple, clinically relevant, accountable, replicable and verifiable.[6]

Over

The objectives of this study were 1) to identify the main available classification systems for CS through a systematic review of the literature, and 2) to analyze qualitatively and compare the advantages and deficiencies of each system through a pre-defined comparative framework based on criteria recognized as important by an international panel of experts.

## Methods

This study has two components: 1) an enquiry to experts about critical characteristics of a classification for CS, and 2) a systematic review of the literature to identify and critically appraise available classifications.

### 1) Questionnaire to panel of experts

A panel of 46 multidisciplinary international experts were contacted by email or personally and asked to collaborate with this study by answering a questionnaire on classifications of CS (Figure S1). They were asked to grade a total of 18 proposed characteristics of a classification system for CS from 1 to 9 (1 = not important; 9 = essential). These characteristics were divided into four main domains (See Table 1): i) General characteristics, ii) Requirements, equipment, necessary skills, iii) Use, and iv) Number and content of categories. Their answers were tabulated in an Excel spreadsheet and ranked according to frequency. Results from this analysis provided the basis for the data-extraction form and assessment of each classification.

**Table 1:** Questionnaire on characteristics of classifications for caesarean sections: grade given by experts.*

I. General characteristics	Grade#
1. Easy to understand	8.5 (1.2)
2. Categories clearly defined and unambiguous	8.6 (0.8)
3. Categories mutually exclusive	7.3 (1.9)
4. Categories totally inclusive	6.9 (2.8)
5. Categories identifiable prospectively	7.6 (1.5)
6. Reproducible and consistent	8.6 (0.6)

II. Requirement, equipment, skills	
7. Guidance on how to deal with cases that have missing information	7.7 (1.4)
8. Implementation possible without sophisticated equipment/hardware/software	7.7 (1.8)
9. No extensive training/education needed for data collection	6.9 (2.3)
10. Information necessary for classification easy to obtain/readily available	8.0 (1.5)
11.Minimal burden of data collection	7.6 (1.5)
12. Capable of being locally adapted/modified	6.9 (2.5)

III. Use	
13. Can be incorporated in routine data collection systems (e.g. billing and accounting)	7.4 (1.9)
14. Useful for clinicians	8.3 (1.6)
15. Useful for epidemiologists, public health specialists & researchers	8.4 (0.9)
16. Useful to change clinical practice	8.5 (0.7)
17. Useful at local and national level	8.2 (1.2)
18. Applicable worldwide	8.0 (1.5)

*Each item was rated from 1 to 9 (1 = not important; 9 = essential).

# Mean (standard deviation).

### 2) Systematic Review

#### Types of studies

Any study that described a theoretical or practical (i.e. actually tested in patients) CS classification system or model was eligible for inclusion in this review, regardless of the level (e.g. facility, regional, national) in which it was applied. We included studies regardless of whether or not the main purpose of the manuscript was to propose a classification (i.e. the classification could be a secondary outcome in the study).

#### Type of participants

Only studies presenting CS classification systems for low-risk or unselected/general obstetric patients were included.

#### Type of classification systems

Any type of CS classification system described in sufficient detail to be understandable and replicable was accepted. Any system or model that systematically grouped or organized CS, obstetric populations or other items (traits, characteristics, variables, attributes) potentially related to the performance of CS into categories was considered a classification. Whenever a classification was presented in more than one publication, data were extracted initially from the original source and complemented, if necessary, with information presented on subsequent publications that reported on its use.

#### Search strategy for identification of studies

Three electronic databases were searched (MEDLINE, EMBASE and LILACS) for articles published from inception to November 26 2008. The search strategy used the following general terms, expanded and adapted for each database: "classification" or "taxonomy" or "nomenclature" or "terminology" and "cesarean section" or "cesarean delivery" or "abdominal delivery" (exact terms presented in Figure S2).

There were no language or country restrictions. Classic review articles, textbooks and published letters were also examined for potentially eligible studies. We checked the references of all articles chosen for full-text evaluation. Experts were contacted and emails sent to authors of potentially eligible studies, inquiring about details, unpublished material and their knowledge of other relevant studies on CS classification.

#### Screening and data extraction

All citations identified were downloaded into Reference Manager® software version 10. The citations were organized and duplicates deleted. Two investigators (MRT and APB) independently screened the results of the electronic searches to select potentially relevant citations based on title and abstracts, according to the criteria defined above. Discrepancies were resolved through consensus. When a citation was considered relevant or when title/abstract was deemed insufficient for decision on inclusion/exclusion, the full texts were retrieved and evaluated.

All articles selected at first screening were read and abstracted individually by the two reviewers using a structured data-extraction form specifically created for this review (Figure S3). Data extracted were compared and discussed by the two reviewers and a final extraction form was compiled. Information extracted from each article included: 1) main purpose of classification, 2) type of study (theoretical versus clinical), 3) characteristics of study and site (setting, CS rate, number of cases, inclusion/exclusion criteria), 4) general characteristics of the system, 5) requirements and skills for implementation, 6) potential use of the classification, 7) specific characteristics of the classification, 8) main strengths and weaknesses of the system reported by the authors, and 9) main strengths and weaknesses of the system as per reviewers. When data in the original publication were not sufficiently detailed, authors were contacted for additional information. In order to assure consistency in the assessment of the classifications over time, the reviewers compared newly extracted with previously extracted articles and forms.

#### Semi-qualitative evaluation of classifications

A general comparison table was constructed describing the main characteristics, strengths and weaknesses of each classification system. Seven specific domains (ease of use, clarity, exclusiveness of categories, inclusiveness of classification, possibility of using classification prospectively, reproducibility and requirements for implementation) were graded (2 = good; 1 = median; 0 = poor).The final grade of each classification ranged from 0 to 14, the higher the grading the better the classification. Each classification was assessed and scored independently by the two reviewers, the answers were compared and discussed until a consensus was reached.

To assess each classification beyond a theoretical model, we created a set of 12 different clinical case-scenarios (Figure S4). After reading and extracting data from each classification system, the two reviewers independently tested the classification using these 12 clinical cases. As opposed to the data extraction, the results of these case scenarios were not compared, reviewed or discussed between the reviewers since we aimed to assess inter-rater agreement. Performance of each classification was assessed by: a) the agreement between the two reviewers in classifying each case in one of the proposed categories (reproducibility); b) the possibility of including each of the 12 cases in no more than one of the categories proposed by the classification (exclusiveness); and c) the ability to include each of the 12 cases into a specific category (inclusiveness).

## Results

### 1) Questionnaire to panel of experts

Of the 46 experts contacted, 38 returned the questionnaire on CS classifications (82% response rate). For each of the first three domains: (i) general characteristics, (ii) requirements, equipment and skills, and (iii) use of the classification, the median grade was either 8 or 9 (over a maximum score of 9). Table 1 presents the average grade given to each of the questions in these domains. According to the experts, a CS classification should provide clearly defined and unambiguous categories, the data needed should be easy to obtain and it should be useful to help change clinical practice. Two-thirds of the experts (25/38) answered that ideally, a classification should have "between 6 and 10" main categories, while the rest suggested "5 at the most" (data not shown).

### 2) Systematic Review

**Figure 1:**
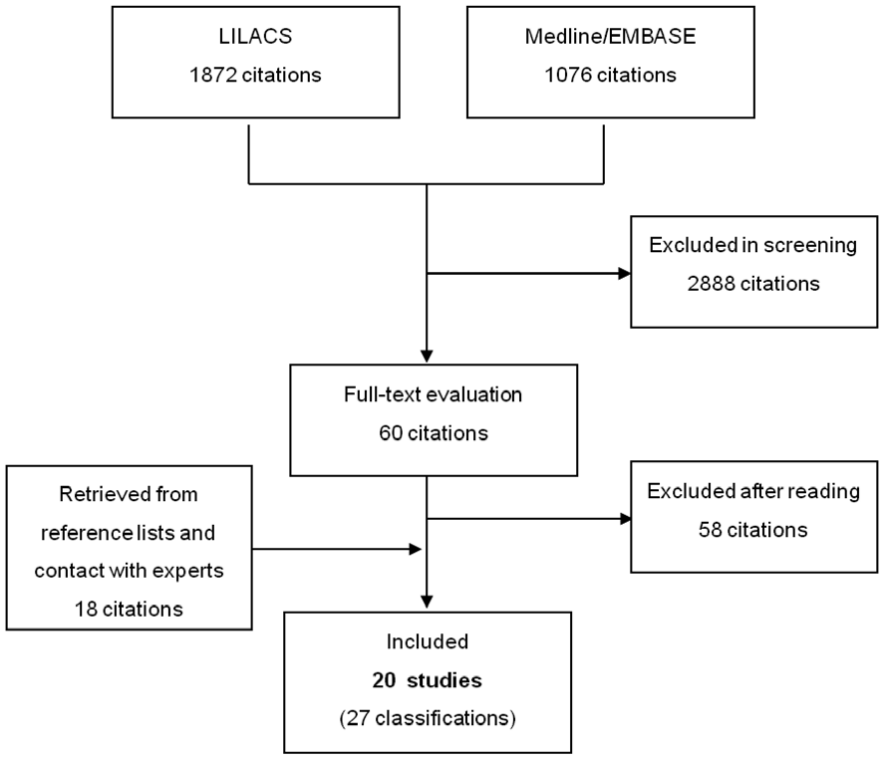
Flow chart of the process of identifying and selecting classifications.

The search strategy yielded 1076 citations in the Medline and EMBASE and 1872 in LILACS. A total of 60 were selected for full-text evaluation (Figure 1). A total of 20 relevant studies were retrieved and 27 different classifications were included (Table 2); one study[4] presented three classifications and two studies [13], [14] presented two classifications each. These 27 classifications were grouped into 4 general types, according to the main unit being classified: indication (N = 12),[4], [7], [15]–[24] degree of urgency (N = 5),[13], [14], [25], [26] woman characteristics (N = 4)[6], [27]–[29] and other systems (N = 6).[4], [13], [30]–[32] Table 2 presents the main characteristics and performance of the 27 classifications, the overall score obtained and the results of the 12 case scenarios.

**Table 2:** Main characteristics of 27 classifications for caesarean section and results from the 12 case-scenarios.

	Main Characteristics*								Case-scenarios (N = 12)^#^		
Classifications	Easy^1^	Clarity^2^	Mutually exclusive^3^	Totally inclusive^4^	Prospective Identif. categories^5^	Reproducibilty^6^	Implementability^7^	Overall score (max. 14)	% disagreement between raters	% cases classified in >1 category	% cases not included in any category
**Indication based**											
Althabe 2004 [15]	2	2	0	2	1	1	1	**9**	17	8	8
Anderson 1984 [7]	2	0	2	2	0	1	2	**9**	8	8	0
Calvo 2009 [16]	2	2	0	2	1	0	1	**8**	58	58	0
Prytherch 2007 [22]	2	2	0	0	1	0	2	**7**	33	8	58
RCOG 2001 (a) [4]	2	0	0	2	1	0	2	**7**	42	50	0
NICE 2004 [20]	2	1	0	0	1	1	2	**7**	-	-	-
Gregory 1994 [18]	1	0	2	0	1	1	1	**6**	25	8	0
Nico 1990 [21]	1	1	0	2	1	0	0	**5**	83	17	0
Stanton 2008 [23]	2	0	0	0	1	0	2	**5**	50	58	8
Unmet needs network 2000[24]	2	0	0	0	1	0	2	**5**	42	28	33
Cisse 1998 [17]	1	0	0	0	1	0	2	**4**	83	42	42
Kushtagi 2008 [19]	1	0	0	0	0	0	1	**2**			
**Degree of urgency based**											
Van Dillen 2009 (a) [14]	2	0	2	2	1	0	2	**9**	33	0	0
Nicopoullos 2003 (a) [13]	2	1	1	2	0	0	2	**8**	67	42	8
Lucas 2000 [26]	2	0	1	2	0	0	2	**7**	58	42	0
Van Dillen 2009 (b) [14]	2	0	2	0	1	0	2	**7**	17	0	17
Huissoud 2009 [25]	2	0	2	0	0	0	2	**6**	50	17	25
**Women based**											
Robson 2001 [6]	2	2	2	2	2	2	2	**14**	0	0	0
Denk 2006 [28]	2	2	2	2	2	2	1	**13**	8	8	0
Cleary 1996 [27]	2	2	2	0	2	2	2	**12**	8	0	0
Lieberman 1998 [29]	1	0	0	1	1	1	1	**5**	33	25	0
**Other types**											
RCOG 2001 (b) [4]	2	1	0	0	2	1	2	**8**	-	-	-
RCOG 2001 (c) [4]	2	1	0	0	1	1	22	**7**	-	-	-
Nicopoullos 2003 (b) [13]	2	1	-	0	-	1	1	**5**	-	-	-
ICD 10 1992 [32]	1	0	1	2	0	0	1	**5**	50	8	0
WHO 2004 [30]	2	0	0	2	0	0	1	**5**	42	8	0
Guidotti 2008 [31]	2	0	0	0	1	0	0	**3**	-	-	-

Code: 2 = good,  = regular, 0 =  poor, ; -  =  not applicable.

1-Easy: how much effort or time it takes to understand main concepts, logic and rules of the classification.

2-Clarity: clear, objective, precise and unambiguous definitions given for each category.

3- Mutually exclusive: each unit being classified by the system (e.g. woman or CS) can only be placed in a single of the existing categories.

4- Totally inclusive: Each and every unit being classified can be placed in at least one of the categories.

5- Prospective identification of categories: allows classification of the patient into one of the categories before she is taken to the operating theater.

6- Reproducibility: probability that the same case would be classified in the same category by different raters.

7- Implementability: human and material requirements needed to introduce and maintain the classification in continuous use.

Table 3 shows the main general strengths and weaknesses of each the 4 general types of classifications. Outlines of each of the 27 classifications are provided in Figure S5.

**Table 3:** Main types of Classification Systems for cesarean section: general strengths and weaknesses.

Name and main question	Strengths	Weaknesses
**Indication**WHY	Information usually routinely collected in any maternity, therefore it is easy to implement.Allows to look at the contribution of:• maternal vs fetal indications• absolute vs relative indications	No clear uniform definitions for common indications (e.g. fetal distress, failure to progress, dystocia).Poor reproducibility unless clear diagnostic definitions are given and rules on hierarchy of classification (for cases with >1 indication)Categories are not mutually exclusive (could be >1 primary indication)Not totally inclusive (unless large number or "Other indications" category exist)"Other Indications" category makes data analysis difficultNot very useful to change clinical practice
**Degree of urgency**WHEN	Conceptually easy, almost intuitiveCould improve communication between professionals (obstetricians, anesthesiologists, nurses) and ultimately improve maternal-perinatal outcomes	Does not provide clear definitions for each of the categoriesPoor reproducibility unless clear definitions are given and staff is trainedCut-offs proposed (time to delivery) are subjective and not evidence-based.Not very useful to change clinical practiceLimited utility for policy makers, epidemiologists, public health specialists
**Patient characteristics**WHO	Conceptually easy and clearly defined categoriesInformation routinely collected in most maternities, easy to implementMutually exclusive and most are totally inclusiveGood reproducibilityProspective, allows modifications in clinical practiceTested in different countries and in large datasets	Does not look at the reason for performing CS on that womanThe case-mix ones are not totally inclusive; they analyze only a portion of all women delivering by CS at a facility
**Other systems**WHERE, HOW,BY WHOM and combinations	Address important but neglected details often overlookedthat could compromise clinical outcomes and should receive more investmentOffer valuable info for administrators and policy makers	Some need adjustment, improvement, clearer definitionsSeveral are just theoretical models and have not been tested in real lifeSome of the data required not usually collected in most maternities; would require some effort to be implemented; limited utility for clinicians

#### Indication based classifications

Table 4 presents the main details of the 12 classifications that belong to this category. Four [19], [20], [23], [24] of the twelve indication classifications presented only theoretical models. The other eight classifications[4], [7], [15]–[18], [21], [22] were tested on actual patients in studies with sample sizes ranging from 498 to 454,668 deliveries and CS rates from 0.6 to 25%. Only three classifications [15], [16], [22] provided clearly defined and unambiguous categories. For example, Althabe et al[15] proposed a CS classification along with a guideline containing specific, precise and clear definitions for indications such as dystocia, acute intrapartum fetal distress and several maternal indications, whereas Anderson's[7] classification also used these same terms but did not provide any details or parameters on how to decide that this was indeed the indication for the CS. Therefore, Althabe's classification was considered clear in its definition of categories, while Anderson's was considered unclear. On the other hand, Anderson's classification provided clear hierarchical rules on how to classify a woman with more than one indication for CS (for e.g. a case with previous CS and dystocia), while Althabe's classification did not provide instructions on how to deal with such cases, which could theoretically be classified in more than one category. This lead us to grade the system proposed by Anderson as being mutually exclusive, while Althabe's classification scored poorly on this characteristic. Only two classifications offered mutually exclusive categories[7], [18] and five were totally inclusive.[4], [7], [15], [16], [21], meaning that each and every possible indication could be placed in at least one of the categories provided by the authors. Over half of these classifications were judged easy to implement.[4], [7], [17], [20], [22]–[24] None of the classifications allowed prospective identification for all categories and in two classifications[7], [19] less than half of the categories could be prospectively identifiable. This refers to the possibility of including a woman into one of the existing indication categories provided by the authors before she is actually taken to the operating theater. Two classifications, Althabe and Anderson's[7], [15] obtained the best overall grade for this group of classifications (9 out of a maximum of 14 points).

**Table 4:** Classifications for caesarean sections based on indications.

Author, year, name	N of major/subcategories: main categories	Special Characteristic
**Althabe, 2004**, Mutually Exclusive Clinical Indication System for Non-emergency CS	**8/0**: Extreme emergency, previous CS, dystocia, intrapartum acute fetal distress, podalic presentation, maternal causes, fetal causes, Other	Gives detailed definitions and flow charts for most proposed categories (unpublished material obtained from authors). Tested on real patients.
**Anderson & Lomas, 1984**, Causal Model for Indications of CS	**5/0**: Previous CS, breech, dystocia, fetal distress, other	Simple, few and well defined categories. One of the few classifications which gives clear hierarchical decision rules.Tested on real patients.
**Calvo 2009**, Mallorca Multifaceted System for Classification of CS	**2/17**: Prescheduled CS, emergency CS	Good definitions for most indications but may be difficult to implement in developing countries. Tested on real patients.
**Prytherch 2007** Modified Unmet Obstetric Needs Network Classification	**5/6**: Antepartum haemorrhage, malpresentations, Ruptured uterus, cephalo-pelvic disproportion/obstructed labour, >2 previous CS	Simple and short, useful in settings with low CS rates. Covers only CS related to absolute maternal indications. Tested on real patients.
**RCGO, 2001**, Primary indications for CS	**21** 1 **/0**	Relatively easy but lacks clear definitions in some categories and hierarchical rules for classifying cases with >1 indication. Tested on real patients.
**NICE, 2004** Evidence based planned CS	**8/0**: Breech, multiple pregnancy, preterm birth, SGA, PP, cephalopelvic disproportion in labour, mother-to-child transmission of maternal infections, maternal request	Incomplete. Could help to evaluate degree of adherence to evidence-based recommendations in different settings. Not tested on real patients.
**Gregory, 1994** Indications for repeat CS	**5/21**: Breech, dystocia, fetal distress, elective repeat CS, other	Conceptually easy. Tries to analyse and compare elective repeat CS versus repeat CS for medical reasons. Tested on real patients.
**Nico, 1990**, CS indications with dystocia or not.	**3/6**: Programmed CS, not programmed CS but not due to failure to progress, CS for failure to progress or dystocia	Gives clear definitions for several types of dystocia, an important indication for CS. Tested on real patients.
**Stanton, 2008** Absolute and non-absolute maternal indications for CS	**2/13**: Absolute maternal indications (hierarchical), Non absolute indications (non hierarchical)	Conceptually easy to understand and useful for developing countries. Could improve if more detailed definitions were given for each of the categories, along with examples. Not tested on real patients.
**Unmet Needs Network, 2000** Absolute maternal indications for CS	**4/13**: Malpresentations and malpositions, antepartum haemorrhage, maternal diseases, fetal reasons	Conceptually easy, useful from public health, helps detect underuse of CS. Clearer definitions of categories would improve reproducibility. Tested on real patients.
**Cisse 1993**, Senegalese 3 groups of indications for CS	**3/9**: Obligatory, prudent, necessary	Relatively easy, meaningful in countries/settings with very low CS rates. Clearer definitions of categories would improve reproducibility. Tested on real patients.
**Kushtagi 2008**, Documentation of indication for delivery and for CS	**2/5**: Indication for termination, Indication for CS	Simple and easy, focuses on conceptual distinctions in trying to understand reasons that lead to CS. Not tested on real patients.

CS: Caesarean section, NICE: National Institute for Clinical Excellence, PP: Placenta praevia, RCOG: Royal College of Obstetricians and Gynecologists, Subcat: Subcategory, VD: Vaginal delivery.

1. Breech, malpresentation/unstable lie, multiple pregnancy, presumed fetal compromise, cord prolapse, chorioamnionitis, other fetal, PP actively bleeding, PP not actively bleeding, antepartum/intrapartum haemorrhage, placental abruption, pre-eclampsia/eclampsia, maternal medical disease, failure to progress (induction/in labour), previous CS, uterine rupture, maternal request, previous poor obstetric outcome, previous physically or emotionally traumatic VD, previous infertility, other maternal.

#### Urgency based classifications

Table 5 presents the main characteristics of these classifications. All five classifications based on degree of urgency had been tested in real life, in studies with sample sizes ranging from 18 to 407 cases in settings with CS rates ranging from 17.7% to 27%. All were judged easy to understand and implement. Three had mutually exclusive categories,[14], [25] and two were totally inclusive.[13], [14],[26] None of the classifications allowed prospective identification for all categories and in two, less than half of the categories proposed could be prospectively identifiable. Van Dillen's classification[14] obtained the best overall grade (9 out of a possible maximum of 14) (Table 2).

**Table 5:** Classifications for caesarean sections based on degree of urgency.

Author, year	Major categories/subcategories: Description of major categories	Special Characteristic
**Van Dillen 2009 (a)** Urgency of CS classification based on clinical definition with interpretation	**4/0**• 1: immediate threat to the life of mother or fetus• 2: Maternal or fetal compromise but not immediately life threatening• 3: The mother needs early delivery but there is no maternal or fetal compromise• 4: Delivery timed to suit the mother or the staff	Relatively easy and an improvement over simple binary classification. Could improve if more detailed definitions were given for each of the categories, along with examples. A total of 79 doctors tested it on 18 theoretical case-scenarios.
**Nicopoullos 2003**, Priority of delivery by CS	**4/0**• Crash (10–20 min)• Urgent (up to 30 min)• Emergency (up to 2 h)• Elective (no time limit)	Simple, few and well defined categories. However, offers no evidence to support the cut-offs proposed for each category. Tested on real patients.
**Lucas 2000**, Urgency of CS classification based on clinical definition	**4/0**• emergency• urgent• scheduled• elective	Same as Van Dillen but with less definitions and guidelines for use. Conceptually easy but needs to exemplify better the clinical situations that would be classified under each category. Tested on real patients.
**Van Dillen 2009 (b)** Traditional Binary System for Degree of Urgency	**2/1**• 1ary: if vaginal delivery was not intended• 2ary: if vaginal delivery was attempted	Simple and easy, but offers very limited amount of information. Could improve if more detailed definitions were given for each of the categories, along with example. A total of 79 doctors tested it on 18 theoretical case-scenarios.
**Huissoud, 2009**Color codes for emergency CS	**3/0**• green: non-urgent CS (up to 1 h interval)• yellow: urgent (<30 minutes)• red: extremely urgent (<15 minutes)	Conceptually easy and simple. Could improve communication between staff and ultimately improve maternal and perinatal outcomes. Tested on real patients.

#### Woman-based classifications

Table 6 presents the main characteristics of all four women-based classifications. These were tested in real life, with samples ranging from 2876 to 222,013 births, in settings with CS rates ranging from 7.9% to 31%. Three classifications presented mutually exclusive categories,[6], [27], [28] two were totally inclusive,[6], [28] and two were judged very easy to implement.[6], [27] Although the 10-group (Robson's) classification[6] received the maximum grade in this type of classification, the 8-group (Denk)[28] and the case-mix (Cleary)[27] classifications also obtained high grades (Table 2).

**Table 6:** Classifications for caesarean sections based on women's characteristics.

Author, year	Major categories/subcategories: Description of major categories	Special Characteristic
**Robson 200**1,The 10 group system.	**10/0**1: Nulliparous, single cephalic term, spontaneous labour2: Nulliparous single cephalic term, induced or CS before labour3: Multiparous no previous scar, single cephalic term, spontaneous labour4: Multiparous no previous scar, single cephalic term, induced or CS before labour5: All multiparous 1 or more previous scar, single cephalic term6: All nulliparous, single breech7: All multiparous, single breech including those with previous scars8: All multiple pregnancies including those with previous scars9: All singleton pregnancies in transverse or oblique lie, including women with previous scars10: All women with single cephalic preterm pregnancy, including women with previous scars	Conceptually easy, clearly defined categories that are totally inclusive, mutually exclusive; little room for misunderstanding or misclassification. All info is easily available from medical records. Could be easy to implement in both high and low resource settings. Prospective classification allows for changes in clinical management. However, does not specify reason for CS. Tested on real patients.
**Denk 2006**,8 group system	**8/24**Primary CS:• Standard nullipara,• Standard multipara,• Malpresentation nullipara• Malpresentation multipara,• All multiple gestation,Singleton pretermRepeat CS:• Standard with prior CS,• All other with prior CS	Same as Robson 2001. The idea of separating 1^ary^ from repeat CS is simple and may have benefits. Tested on real patients.
**Clearly 1996**, Standard primipara	**1/0**White, 20–34 year-old, height >155 cm, with singleton cephalic fetus >37 weeks, in the unit at which she originally booked, excluding cases with pre-existing diseases or complications of pregnancy.	Conceptually easy, well defined parameters. Analyzes a specific group of women that represent a large fraction of the population delivering in most maternities. Not totally inclusive and definition is very regional (e.g., it would be irrelevant for African countries). Would therefore need to be adapted to different settings. Tested on real patients.
**Lieberman 1998**,Case mix model for adjusting CS rates	**3/18**• Nullipara• Multipara with no previous CS• Multipara with one or more previous CS.	It proposes a matrix, mixing women's characteristics and some indications. Would allow fair comparisons between facilities of different levels. However, requires a step of “standardization” which involves statistical expertise and software. Tested on real patients.

#### Other types of classifications

The six other types of classifications are presented on Table 7. One of these classifications was just a theoretical model that was not tested in real life;[31] the other five were tested in studies involving from 137 to 32,222 cases in settings with CS rates ranging from 23% to 35%. These classifications proposed from 3 to 21 main categories and up to 39 subcategories. None of these classifications had mutually exclusive categories and two were totally inclusive.[30], [32]

**Table 7:** Other types of classifications for caesarean sections.

Author, year	Major categories/subcategories: Description of major categories	Special Characteristic
**RCOG 2001**, CS according to organizational and staffing factors	**5/0**Size of maternity unit, presence of neonatal intensive care unit, being a tertiary referral center, affiliation with a medical school, availability of 24-hour anaesthetist	Looks at important factors generally overlooked Easy system with clear and well defined categories; data easily available at most settings. Could be useful to compare similar settings as to rates of CS, indications or types of patients. Tested on real patients.
**RCOG 2001**, Potentially complicated CS	**7/0**PP, placental abruption, at full cervical dilation, in obese women, for preterm delivery <32 weeks, for multiple pregnancy, in women with multiple previous CS	Simple and easy to implement. Could be useful to audit quality/quantity of human resources available in different settings and over time and see how this impacts maternal and perinatal morbidity and mortality. Tested on real patients.
**Nicopoullos 2003**, Documentation of obstetric care in caesarean section	**18/0**Indication, name of surgeon, grade of surgeon, name of assistant, name of anaesthetist, type of anaesthetic, skin incision time, skin incision type, surgical findings, uterine incision type, engagement of presenting part, fetal delivery, placental delivery, uterine cavity check, presence of paediatrician, adnexal check, estimate of blood loss, post-op care plan.	It tries to standardize the documentation on CS. Important as a legal instrument in cases of litigation and allows auditing and improvement of care. Tested on real patients.
**World Health Organization 1992**, ICD 10 classification	**7/0**Single delivery by elective CS; single delivery by emergency CS; single delivery by cesarean hysterectomy; other single delivery by CS; single delivery by CS, unspecified; multiple delivery, all by CS; other multiple delivery by combination of methods	Few categories, therefore simple, easy and quick to fill in, well known internationally. However it is of limited clinical relevance. Tested on real patients.
**Villar 2004**,Global Survey classification	**3/0**Elective CS, no labour; emergency CS, no labour; intrapartum CS	Simple and easy, but offers very limited amount of information. Could improve if more detailed definitions were given for each of the categories, along with examples. Tested on real patients.
**Guidotti 2008,**Safety of CS in resource poor settings	**12/39**Necessity for a CS, maternal condition, fetal condition, surgical team, surgical procedure, anaesthesia procedure, surgical Instruments, anaesthesia equipment, operative theater conditions, drugs, maternal post-operative care, neonatal post-operative care	Conceptually easy. Takes into account other elements beyond indication that can affect outcomes of CS. However, since necessary data is not routinely collected, it would require some effort and training to implement. Not tested on real patients.

## Discussion

This review identified 27 classification systems which were grouped into 4 general types, based on the main unit being classified. Classifications based on **indications for CS** were the most frequent type. The main question answered by this type of classification is "why" the CS was being performed, an information routinely registered and available in any maternity, therefore making this type of system easy to implement in any setting. On the negative side, almost all of the models in this group of classifications had categories that are not mutually exclusive and had low reproducibility. Due to these main drawbacks, the disagreement between reviewers in the case-scenarios was high (see Table 2); in six classifications there was disagreement in at least 6 of the 12 case-scenarios. Main weaknesses of these systems include: a) poor/unclear definitions for some of the most common conditions that lead to CS (e.g. dystocia, fetal distress) and therefore questionable inter-rater reproducibility; b) categories not mutually exclusive, implying that there would need to be some kind of hierarchy guideline to classify cases with >1 primary indication; c) not being totally inclusive, unless an extensive list of indications is provided or an “Other indications” category is created; and d) not be very useful to change clinical practice, since most of the indications are not prospectively identifiable (Table 3). This type of classifications proposed the largest number of categories, although some, such as Anderson's[7] were quite simple. This specific classification, together with Althabe's had the highest rating in this group. Unlike others in this group, Anderson's classification was judged easy to understand and implement, had a good inter-rater reproducibility and was all inclusive. A unique asset of this model was that it presented clear hierarchical rules for classifying cases with >1 indication for CS which made the categories mutually exclusive.

Classifications based on **degree of urgency for CS** were also theoretically easy to understand and implement due to the reduced number of categories proposed (Table 2). This type of classification, which basically answers "when" (or how quickly) the CS should be performed, could improve communication between health professionals (nurses, obstetricians, anesthesiologists) thus potentially lead to better maternal and perinatal outcomes. A weak point of several of these classifications is the lack of clear and unambiguous definitions for each of the proposed categories, which could compromise inter-rater reproducibility, comparability and interpretation. Three of the five presented 50% or more of disagreement between reviewers in the case-scenarios. Additionally, the cut-offs (time to delivery intervals) proposed to define each category are subjective and not evidence-based. Finally, the amount of information provided by these systems is very limited and therefore this type of classification would have to be complemented by other types, in order to be more useful.

Classifications based on **woman characteristics** basically tell us "who" is being submitted to CS, based on maternal and pregnancy characteristics. These represented 4/27 systems identified. Most of these classifications are conceptually easy and simple, have relatively few, and clearly defined categories which are mutually exclusive and allow cases to be prospectively identified upon admission, which could be useful to change clinical practice. Due to all these characteristics, these classifications could be easily implemented and would be highly reproducible as shown with the high agreement in the case scenarios (Table 2). Although most of these classifications are totally inclusive, the case-mix types are not since they only assess CS in a subgroup of women with a specific set of predefined characteristics, such as Cleary's[27] "standard primipara". Robson's 10 group,[6] along with Denk's 8 group[28] classifications got the highest overall theoretical ratings and also performed very well on the practical case scenarios.

**Other types of classifications**, which represented 6/27 classifications, address questions such as "where" the CS is being performed, "by whom", "how" (under what conditions and circumstances) or combinations of questions. By focusing on aspects often overlooked by other classifications, these systems provide administrators and with useful information about aspects that could affect maternal and perinatal outcomes and perhaps need more attention and investment. However, some of these classifications would need improvement and clearer definitions of some categories. Moreover, several of these systems are only theoretical and have never been tested in real setting. Since some of the data required are not usually collected in most maternities, these systems would require some effort and time to be implemented and not all items in these classifications will be relevant or applicable in all settings.

Based on the methodology used in this systematic review, Anderson's[7] and Althabe's[15] classifications obtained the highest grades and the best performance for indication-based classifications. This can be attributed to the fact that these two classifications provide very clear definitions of categories and precise decision rules or hierarchy on how to classify a case with >1 indication into a single specific category. In the degree of urgency systems, Van Dillen 2009a[14] was the best rated classification. Robson's 10-group model[6] was in first place among the women-based classifications and obtained the highest overall grade and best performance on the case-scenarios.

Each of these classifications offer intrinsic advantages and disadvantages and could be considered more or less useful depending on the objectives of the user. The two classifications with the best overall scores in this group (Robson and Denk [6], [28]) are easy to understand, clear, mutually exclusive, totally inclusive, reproducible and allow prospective identification of categories. Additionally, they offer flexibility to adapt to different clinical settings, important aspects if one wishes to implement modifications in clinical protocols to decrease or increase CS rates. Robson's classification offers the possibility of subdividing three of its main categories into subcategories. Namely women at term with a singleton, cephalic, term fetus being submitted to a CS either after induction (groups 2a-nulliparas and 4a-multiparas) or electively (groups 2b-nulliparas or 4b-nulliparas), and women with either one or more than one previous CS (group 5a and 5b, respectively). These subdivisions would provide important information and help to understand differences between different settings or at the same setting over time, in these 3 categories. Despite the fact that the “10-group classification” would actually become a “13-group classification”, these subdivisions do not add any substantial amount of work since the information needed is routinely available in maternal charts. A problem with the women-based classifications is that they do not present why (indications) or when (degree of urgency) the CS was performed, which are also important aspects.

After a thorough and careful analysis of a large number of classifications and systems for ceasarean deliveries, we acknowledge the fact that, at the present, there is no single ideal classification for all settings and that would fulfill the expectations and needs of every health professional. The choice of a specific classification will depend on the main objectives of the professionals who are going to use it. However, given the flexibility of some of the existing classifications, we believe it would be possible to create a hybrid model based on the woman-characteristics system with additional layers of other classifications for each of the individual categories proposed in the woman's classification. For instance, Van Dillen[14] and/or a modified version of Anderson's indication system[7] could be used within each of the 10 (or 13) categories proposed by Robson[6] or the 8 categories proposed by Denk.[28] This would allow comparison of degree of urgency for CS as well as indications in a homogeneous group of women, for example multiparas at term in spontaneous labor with a singleton cephalic fetus (Robson's group 3a), which represent a large proportion of all deliveries in any setting.

This systematic review had several strong points, starting with its uniqueness. This is the first study specifically designed to retrieve, analyze and critically appraise existing classifications for CS. We developed a broad search strategy, in order to capture the largest possible number of publications on this topic. We tried to reduce bias by using a panel of experts to determine what variables to analyze and two independent reviewers to extract data and test each classification in practical case-scenarios.

Potential limitations included difficulties in retrieving articles through electronic databases, possibly due to the lack of appropriate keywords to index this topic. We also acknowledge the possible existence of other unpublished CS classifications that could not be located, despite efforts to contact experts. Additionally, despite the use of strict methodology and double data extraction at all steps of the systematic review, there is always potential for subjectivity in the semi-qualitative assessment of the classifications. We also acknowledge that the scoring system presented on Table 2 may have limitations. To the best of our knowledge, there are no validated tools for assessing the characteristics of any classification system. This led us to create such a tool, which we tried to keep as simple and objective as possible. However, the use of only three possible grades for each of the domains of the classifications, although straightforward and easy, may be questionable.

Overall, we detected a basic need for clear, unambiguous and precise definitions for common obstetrical diagnoses and terms used to define categories in many of the classifications. Standardization of these terms is an essential step to improve inter-rater reproducibility and allow consistent and reliable comparison of information at the same setting over time and between different settings at various levels (local, regional and national). Specifically, in the indication classifications, terms/diagnoses such as fetal distress, dystocia, failure to progress, cephalo-pelvic disproportion, obstructed labor, macrosomia, failed induction and failed trial of labor would need to be more clearly defined using unambiguous and preferably evidence-based terminology. Furthermore, it would be preferable to avoid the need for sophisticated equipments or technology (such as electronic fetal monitors or scalp pH) not routinely available in low-resource settings. Despite a few discrepancies, the terms and definitions used in the degree of urgency classifications (for e.g. urgent, emergency, crash, scheduled and elective) tend to be more precisely defined but none of them are evidence-based. Therefore, there is a need to conduct studies that assess if there are any significant differences in maternal and perinatal morbidity and mortality according to the time interval between decision to incision (or actual delivery). Only then would it be possible to establish more precise cut-offs used to define each of these categories.

In the context of international recognition of the difficulties in understanding and controlling the increase and inequitable use of CS worldwide, this systematic review suggests that, among all classifications identified, women-based classifications in general, and Robson's classification, in particular, would be in the best position to fulfill current international and local needs, and that efforts to develop an internationally applicable CS classification would be most appropriately placed in building upon this classification. The dissemination and implementation of a single CS classification system will allow auditing, analyzing and comparing rates of CS across different hospitals, cities, countries and regions. With a clear understanding of why, when, where, how and on whom CS are being performed, it would then be possible to propose and implement effective strategies and actions specifically targeted at high-risk groups, and thus possibly reduce or increase the rate of CS in order to continue improving maternal and perinatal outcomes.

## Supporting Information

Figure S1 Survey questionnaire. Questionnaire sent to international panel of experts to rate items considered important in a classificaiton for cesarean sections.
(0.07 MB DOC)

Figure S2 Search Strategy for CS classifications. Search strategy used for systematic review.
(0.03 MB DOC)

Figure S3 Data extraction form used to analyse and critically appraise existing Cesarean section classifications.
(0.08 MB DOC)

Figure S4 Clinical case scenarios. Twelve clinical case scenarios created to test the existing classifications for Cesarean sections.
(0.03 MB DOC)

Figure S5 Outline of the 27 classifications for Cesarean sections included in the systematic review.
(0.20 MB DOC)

PRISMA statement PRISMA statement
(0.06 MB DOC)
