# Supplementary material for: Classifications for Cesarean Section: A Systematic Review
Source: PLoS One. 2011 Jan 20;6(1):e14566. doi: 10.1371/journal.pone.0014566 (PMC3024323; doi:10.1371/journal.pone.0014566)
Supplement: Figure S1 — Survey questionnaire. Questionnaire sent to international panel of experts to rate items considered important in a classificaiton for cesarean sections. (0.07 MB DOC) [file pone.0014566.s001.doc]

**Supporting information 1. Survey Questionnaire**

**WHO Systematic Review on Classifications for**

**Caesarean Section**

**1.1**


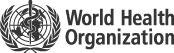


**A. Please score the importance of the characteristics below in a classification for caesarean section from 1 to 9 (1=not important; 9=essential).**

| **I. General characteristics** | **Grade** |
| --- | --- |
| 1. Classification should be easy to understand |  |
| 2. Each part of the classification (category) should be clearly defined and unambiguous |  |
| 3. Categories should be mutually exclusive. Each caesarean section will only belong in one of the categories in the classification |  |
| 4. Categories should be totally inclusive. All caesarean sections will belong in one of the categories of the classification. |  |
| 5. The classification should allow caesarean sections to be classified in such a way that the categories can be identified prospectively. |  |
| 6. Classification should be reproducible and consistent (i.e. different data collectors would classify the same woman (or item) in the same category/group) |  |

| **II. Requirement, equipment, skills** |  |
| --- | --- |
| 7. Classification should give specific guidance on how to deal with cases that have missing information |  |
| 8. Classification should NOT require sophisticated equipment/hardware/software to be implemented |  |
| 9. The person filling in the forms for classification should not require extensive training or education |  |
| 10. Information necessary for classification should be easy to obtain and readily available |  |
| 11. The burden of data collection should be minimal |  |
| 12. Classification should be flexible and able to be locally adapted/modified if needed |  |

| **III. Use** |  |
| --- | --- |
| 13. Classification should be able to be incorporated in routine data collection systems (e.g. billing and accounting services) |  |
| 14. Classification should be useful for clinicians |  |
| 15. Classification should be useful for epidemiologists, public health specialists & researchers |  |
| 16. Classification should be useful to change clinical practice in the future (e.g. if women are classified, they should be able to be classified prospectively so that outcome can be improved in those same group of women in the future) |  |
| 17. Classification needs to be useful at the local as well as at national level |  |
| 18. The classification should be applicable worldwide |  |

**WHO Systematic Review on Classifications for**

**Caesarean Section**

**1.2**


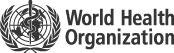


(continuation)

| **IV. Categories: number and content** |
| --- |
| 19. The maximum number of MAIN categories (not including subgroups or sub-categories) in the classification system should be? (select one): 🞎 5 at the most 🞎 6 to 10 🞎 more than 10 |
| 20. In your opinion, which of these variables should be used to create categories of a classification for caesarean section (please select 5):  🞎 maternal age 🞎 parity 🞎 number of previous caesarean  🞎 gestational age 🞎 fetal presentation 🞎 number of fetuses  🞎 degree of urgency (elective, urgency, emergency)  🞎 moment of caesarean in relation to labor (before labor or intra-partum)  🞎 cesarean after induction 🞎 morbidities (mother) 🞎 fetal conditions  🞎 indication for caesarean 🞎 maternal request 🞎 payment mode |
| 21. Please add any other characteristic that you think is important in a classification system for cesarean section and is not listed above: |

**B.1 Do you use a classification for cesarean section in your setting?** No 🞎 Yes 🞎

If you answered “Yes”, please give the name of this classification system and / or details on how to obtain a copy of this system (reference or name of author or contact person, or year of publication):_________________________

___________________________________________________________________________________________

___________________________________________________________________________________________

___________________________________________________________________________________________

**B.2. In your opinion, what is the most useful characteristic of the classification used in your setting?**

___________________________________________________________________________________________

**B.3 In your opinion, what is the least useful characteristic of the classification used in your setting?**

___________________________________________________________________________________________

**C. Please indicate whether you are:**

□Physician □Nurse/midwife □Program manager □Researcher

□other:__________________________________________________

from: □ Africa □ Americas □ Asia □ Europe □ Oceania
