# Supplementary material for: Classifications for Cesarean Section: A Systematic Review
Source: PLoS One. 2011 Jan 20;6(1):e14566. doi: 10.1371/journal.pone.0014566 (PMC3024323; doi:10.1371/journal.pone.0014566)
Supplement: Figure S2 — Search Strategy for CS classifications. Search strategy used for systematic review. (0.03 MB DOC) [file pone.0014566.s002.doc]

**Supporting Information 2. Search Strategy for CS classifications**

**Search performed on Pubmed**

Step 1

("Classification"[Mesh]) or (Classifications) or (Systemat*) or (Taxonomy) or (Taxonomies) or (classification or classif*)

Step 2

("Cesarean Section"[Mesh]) or (Cesarean Sections) or (Delivery, Abdominal) or (Abdominal Deliveries) or (Deliveries, Abdominal) or (Caesarean Section) or (Caesarean Sections) or (Abdominal Delivery) or (C-Section (OB)) or (C Section (OB)) or (C-Sections (OB)) or (Postcesarean Section)

Step 1 and Step 2

**Search performed on EMBASE** ([www.embase.com](http://www.embase.com/))

Step 1

Terminolog* or "nomenclature"/exp or nomenclature or classification/exp or classific* or "category tree" or taxonom* or staging

Step 2

("abdominal operation" AND birth) OR caesarean OR cesarean OR caesarian OR cesarian OR fetectomy OR cesarotomy OR caesarea OR "c-section*" OR "c section*" OR "abdominal delivery" or "abdominal birth" or Postcesarean

Step 1 and Step 2

**Search performed on LILACS**

Step 1

**(**Terminology) OR (terminologia) OR (terminología) OR (Ex V02.310.750) OR (Ex L01.143.506.598.400) OR (Terminology as Topic) OR (Terminología como Asunto) OR (Terminologia como Assunto) OR (Classification) OR (Clasificación) OR (Classificação) OR (Classificacao) OR (Ex L01.100) OR (Ex L01.453.245.275) OR (Etimologia) OR (Nomenclatura como assunto) OR (Compilação) OR (Taxonomia) OR (Sistemática) [Words]

Step 2

(Cesarean Section) OR (Cesárea) OR (Parto Abdominal) OR (Ex E04.520.252.500) OR (Abdominal Delivery) OR (Delivery, Abdominal) OR (Nascimento Vaginal após Cesárea) OR (Parto Vaginal Despues de cesarean) OR (Ex E04.520.252.992) OR (Cesarean Section, Repeat) OR (Recesariana) OR (Cesarea Repetida) OR (Ex E04.520.252.500.150) OR (Repeat Cesarean Section) [Words**]**

Step 1 and Step 2
