# Supplementary material for: Classifications for Cesarean Section: A Systematic Review
Source: PLoS One. 2011 Jan 20;6(1):e14566. doi: 10.1371/journal.pone.0014566 (PMC3024323; doi:10.1371/journal.pone.0014566)
Supplement: Figure S3 — Data extraction form used to analyse and critically appraise existing Cesarean section classifications. (0.08 MB DOC) [file pone.0014566.s003.doc]

**Supporting information 3 - Data Extraction Form**

**WHO Systematic Review on Classifications for**

**Caesarean Section- Data extraction form**


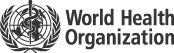


| **A. Identification** | |
| --- | --- |
| 1. ID number (Ref Man) |  |
| 1. Name of 1^st^ author |  |
| 1. Country of study |  |
| 1. Language |  |
| 1. Was this classification published? | No🞎 Yes🞎 Yes and >1 publication🞎 |
| 1. Publication date (or source, if unpublished) |  |
| 1. Study period (if clinical study) |  |
| 1. Name of system (proposed by authors 🞎 or   reviewer´s denomination 🞎) |  |

| **B. Main purpose of the classification** | |
| --- | --- |
| 1. Do authors offer specific guidelines along with the CS classification* ? Yes🞎 No🞎 | |
| 1. Did authors set out to create a classification system for CS as one of their 1^ary^ objective? Yes🞎 No🞎   **If No,** specify 1ary objective and **GO to C:** | |
| 1. Not explicitly stated 🞎 or difficult to understand 🞎 |  |
| 1. MAINLY Clinical (to improve/ modify management) |  |
| 1. MAINLY Public health (official statistics, rates) |  |
| 1. MAINLY Epidemiology (risk factors for CS) |  |
| 1. Other ,specify: |  |

| **C. Type of study** | |
| --- | --- |
| 1. Not explicitly stated – **GO to E** |  |
| 2. Theoretical model only (not actually tested in real life, with cases) – **GO to E** |  |
| 3. Theoretical + practical model (reports data on real cases or patients) – **GO to D** |  |

| **D. Clinical Characteristics of Study** |
| --- |
| 1. Setting where test was carried out (number of hospitals, name of city (ies), name of country (ies): |
| 2 .Total number of cases (patients, cases) analyzed* : |
| 3. CS rate in that population: No info 🞎, or: % |
| 4. Number of examiners (doctors, nurses, technicians, etc…) testing the classification: No info 🞎, or: |
| 5. Inclusion criteria of participants: |
| 6. Exclusion criteria of participants: |

**Grades: 0= No, 1= Partially or Unsure, 2= Yes**

| **E. General characteristics** | **Grade** |
| --- | --- |
| 1. Is this classification easy to understand? |  |
| 2. Is each part of the classification (category) clearly defined and unambiguous? |  |
| 3. Categories should be mutually exclusive. Does each item being classified belong in one (and only one) of the categories in the classification? |  |
| 4. Categories should be totally inclusive. Does each and every item being classified belong in one of the categories of the classification? |  |
| 5. Does this classification allow item being classified to be classified in such a way that the categories can be identified prospectively? |  |
| 6.a. Is the classification completely reproducible and consistent (i.e. different data collectors would classify the same woman (or item) in the same category/group)? |  |
| 6.b. If Kappa test provided by authors, report value |  |

| **F. Requirement, equipment, skills** |  |
| --- | --- |
| 1. Is information necessary for classification easy to obtain and typically or routinely collected in labor and delivery rooms of most hospitals? |  |
| 2. Does the classification give specific guidance on how to deal with cases that have missing information? |  |
| 3. Is it possible to implement this classification without sophisticated equipment/hardware/software? |  |
| 4. Is it possible to fill the forms for classification without extensive training or education? |  |
| 5. Is the burden of data collection minimal? |  |

| **G. Use** |  |
| --- | --- |
| 1. Is classification useful for epidemiologists, public health specialists or researchers? |  |
| 2. Is this classification useful to change clinical practice in the future (e.g. if women are classified, they should be able to be classified prospectively so that outcome can be improved in those same group of women in the future)? Just copy answer to E5 |  |
| 3. Is this classification useful at the local as well as at national level? |  |

| **H. Characteristics of Classification** |  |
| --- | --- |
| 1. **MAIN** question being asked: Who 🞎, Why🞎, When 🞎, How 🞎, By whom 🞎, Where 🞎 |  |
| 2. **MAIN** unit being classified: Woman 🞎, Fetus 🞎, Indication for CS🞎, Degree of urgency for CS🞎,  Other (specify): |  |
| 3.a Number of MAIN categories presented in this classification system |  |
| 3.b Number of Subcategories presented in this classification system |  |
| 4. Which of the following categories are represented in this classification system:  1🞎 maternal age 2🞎 parity 3🞎 previous caesarean  4🞎 gestational age 5🞎 fetal presentation 6🞎 number of previous cesarean  7🞎 degree of urgency (elective, urgency, emergency) 8🞎 number of fetuses  9🞎 moment of caesarean in relation to labor (before labor or intra-partum)  10🞎 cesarean after induction 11🞎 morbidities (mother) 12🞎 fetal conditions  13🞎 indication for caesarean 14🞎 maternal request 15🞎 payment mode | |
| 16. List other characteristics mentioned by this system not provided above: | |

**I: Main strengths of the system according to the authors:**

**J. Main weaknesses of the system according to the authors:**

**K. Strengths of the system detected by reviewers** (to be completed after using the system in the case-scenarios):

**L. Weaknesses of the system detected by reviewers** (to be completed after using the system in the case-scenarios):

Observations, comments and specific questions to ask authors ( author contact information: email, address:_____________________________________________________________________)

Date: Reviewer name: __________________________
