# Supplementary material for: Classifications for Cesarean Section: A Systematic Review
Source: PLoS One. 2011 Jan 20;6(1):e14566. doi: 10.1371/journal.pone.0014566 (PMC3024323; doi:10.1371/journal.pone.0014566)
Supplement: Figure S4 — Clinical case scenarios. Twelve clinical case scenarios created to test the existing classifications for Cesarean sections. (0.03 MB DOC) [file pone.0014566.s004.doc]

**Supporting information 4- Clinical case scenarios**

Suppose you performed a Cesarean in these 12 women, how would you classify each of them? Place the name(s) of the corresponding category (ies) proposed by the classification next to each case.

Name of Classification System:

Reviewer´s name and date:

1. A 23-year- old healthy nulliparous woman at 38 weeks with a singleton cephalic live fetus has been in labor for 12 hours. The cervix has been 4 cm dilated for the past 4 hours, her membranes have ruptured spontaneously 8 hours before, liquor is clear and fetal heartbeat is normal. She has been receiving oxitocin and antibiotics for the past 3 hours.

Category:________________________________

2. A 33-year-old hypertensive multiparous woman (Gesta 5, Para 4, all vaginal deliveries) is admitted at 36 weeks with blood pressure (BP) 180 x 110 mm, singleton cephalic live fetus, severely growth restricted, with oligohydramnios and absent end-diastolic flow on Doppler ultrasound. She is not in labor and her cervix is undilated and she has intact membranes. Category:________________________________

3. A 28-year-old nulliparous is admitted with probable pyelonephritis (fever, back pain, > 1.000.000. leukocytes on urine sample) at 32 weeks gestation with a singleton fetus. Her cervix is 8 cm dilated, the fetus is in breech, membranes are intact and the fetal hearbeat is normal. Category:________________________________

4. A 42-year-old nulliparous woman with triplets, the result of assisted reproduction (tri-chorionic, tri-amniotic) presents at 29 weeks because one of fetus has severe growth restriction, oligohydramnnios and reverse-diastolic flow on Doppler ultrasound, with non-reassuring cardiotochography. The other two fetuses are healthy. She has mild pre-eclampsia and gestational diabetes controlled with diet, is not in labor, membranes are intact, her cervix is undilated and the 1^st^ fetus is in cephalic presentation. Category:________________________________

5. A 17-year-old cocaine-addict woman (Gesta 3 Para 2 ,with 1 previous Cesarean) is admitted at 38 weeks with a singleton fetus, based on ultrasound performed at 20 week. She had only 1 antenatal visit, at 20 weeks. On admission her fundal height is 27 cm, she is in labor, 5 cm dilated, cephalic presentation with ruptured membranes (doesn´t know when they ruptured), has moderate vaginal bleeding and the fetus has persistent late decelarations.

Category:________________________________

6. A multiparous woman (Gesta 3 Para 2, with 1 previous Cesarean) in labor at 38 weeks has a prolapsed cord upon spontaneous rupture of membranes. The fetus is transverse, the cervix is 6 cm dilated and the fetal hearbeat is normal. Category:________________________________

7. A multiparous woman (Gesta 5 Para 4, with 4 previous Cesareans ) is booked for elective Cesarean at 38 weeks. She presents at 36 weeks in active labor, with a singleton cephalic fetus, cervix 3 cm dilated, intact membranes and normal fetal heartbeat.

Category:________________________________

8. A multiparous woman (Gesta 3 Para 2, with 2 previous Cesareans) is brought to the emergency room at 34 weeks with signs of hypovolemic shock (pale, pulse 120 bpm, BP 90 x 40) and no fetal heart beat. The fetus is in breech presentation, the uterus is hypertonic and she has no vaginal bleeding. Category:________________________________

9. A 13-year-old nullipara is admitted in labor at 37 weeks, with a live singleton breech fetus, 2 cm dilated, ruptured membranes and liquor heavily stained with meconium. The fetal heartbeat is normal, her BP is 150 x 100, she is confused and has severe facial and ankle edema. A dipstick urine test is positive for proteinuria (++++). In the admission room she has a convulsion. Category:________________________________

10. A 42-year-old healthy multipara (Gesta 3 Para 2, with 2 previous vaginal deliveries) presents at 38 weeks. She is not in labor, her cervix is not dilated , the fetus is cephalic, with an estimated weight of 2900g, normal amniotic fluid and a normal heartbeat. She and her husband are very anxious and demand an elective Cesarean, because their last child, born 8 years before, has severe neurological handicaps attributed to intrapartum asphyxia.

Category:________________________________

11. A 32-year-old healthy multipara (Gesta 2 Para 1,with 1 previous vaginal delivery) is induced at 42 weeks, because of post-term pregnancy with normal amniotic fluid and an estimated fetal weight of 3100g. After 10 hours of oxytocin, contractions are irregular and the cervix remains uneffaced and 2 cm dilated. The fetal heartbeat is normal. Category:________________________________

12. A 38-year-old morbidly obese type 2 nulliparous diabetic is admitted at 39 weeks with suspected fetal macrossomia. Her fundal height is 44 cm and ultrasound-estimated fetal weight is 4700 g. The fetus is singleton and cephalic, she is not in labor, membranes are intact, she is slightly dyspneic, her BP is 150 x 100, positive proteinuria on dipstick (+++) and she has severe ankle edema. Category:______________________________
