# Supplementary material for: Classifications for Cesarean Section: A Systematic Review
Source: PLoS One. 2011 Jan 20;6(1):e14566. doi: 10.1371/journal.pone.0014566 (PMC3024323; doi:10.1371/journal.pone.0014566)
Supplement: Figure S5 — Outline of the 27 classifications for Cesarean sections included in the systematic review. (0.20 MB DOC) [file pone.0014566.s005.doc]

**Supporting Information 5- Outline of 27 Classifications for cesarean section**

**Classifications based on indication**

**Althabe 2004: 7 Mutually Exclusive Clinical Indication System for Non-emergency CS**

| **Category - Guidelines developed** | **Definition** |
| --- | --- |
| 0. Extreme emergency | This includes situations such as maternal haemorrhage; cord prolapse, suspected uterine rupture or any other situation where the attending physician judged that a delay would constitute malpractice. |
| 1. Previous CS |  |
| 2. Dystocia |  |
| 3. Intrapartum Acute Fetal Distress |  |
| 4. Podalic presentation |  |
| 5. Maternal causes |  |
| 6. Fetal causes |  |
| 7. Other |  |

**Anderson 1984: Causal Model for Indications of CS**

| **Category** | **Level of diagnosis** | **Level of therapeutic response to the diagnosis (CS vs vaginal)** |
| --- | --- | --- |
| Previous cesarean |  |  |
| Breech presentation |  |  |
| Dystocia |  |  |
| Fetal distress |  |  |
| Other |  |  |

Model and decision rules:

1. All multiple-diagnosis deliveries in which one of the diagnosis was a previous CS were assigned to the diagnostic class "previous cesarean birth" (in practice, only 5.5 to 6.5% of the diagnoses of previous CS had any other accompanying diagnosis during the period under study);
2. cases having a diagnosis of breech presentation with either dystocia or fetal distress or both were assigned to the diagnostic class "breech" (this recognized breech presentation as a cause of both dystocia and fetal distress);
3. Cases having the diagnoses dystocia and fetal distress were assigned to the diagnostic class "dystocia" (this recognized dystocia as a cause of fetal distress); and

A case was assigned to the diagnostic class "fetal distress" only if none of the other three diagnoses appeared in the abstract. Cases that did not fall into one of the above four classes were classified as "other".

**Calvo 2009: Mallorca Multifaceted System for Classification of CS**

| **Category** | **Subcategory (full definitions provided)** |
| --- | --- |
| Prescheduled CS | 1. Transverse presentation 2. Breech presentation regardless of parity 3. Complete placenta previa 4. Partial placenta previa encroaching on the internal cervical os 5. HIV-positive pregnant women who meet protocol criteria 6. HIV-positive pregnant women with genital warts over a good portion of the soft birth canal 7. Pregnant women with two previous CS 8. Pregnant women with confirmed active genital herpes virus infection 9. Pregnant women who have undergone previous uterine surgery with opening of the endometrial cavity 10. Twin pregnancies, with the first fetus not in cephalic presentation 11. Other maternal or fetal conditions that may have been agreed upon during a clinical session, such as maternal illness, previous CS, or other problems: prepartum elective CS |
| Emergency CS | 1. Risk of fetal distress 2. Failed induction 3. Arrested labor/failure to progress 4. Fetopelvic disproportion 5. Elective cesarean section during labor 6. Other |

**Prytherch 2007: Modified Unmet Obstetric Needs Network Classification**

| **Category (Absolute Maternal Indications)*** | **Subcategory** | **Inclusion** |
| --- | --- | --- |
| Antepartum Haemorrhage | Abruptio placentae  Placenta previa |  |
| Malpresentations | Transverse lie  Brow, face  Shoulder  breech |  |
| Ruptured uterus |  |  |
| Cephalo-pelvic disproportion / obstructed labour |  | Based on partograph with action line crossed by the dilation line (for the purpose of this classification, this indication was intended to replace all other indications like poor progress, dystocia, prolonged labour, etc. |
| >2 previous CS |  |  |

* These conditions were selected not only because their life-threatening nature, but also as they require specific major obstetric interventions which can be verified through health service records.

**RCGO 2001 (a): Sentinel audit: Primary Indications for CS**

|  | **Categories** |
| --- | --- |
| 1 | Breech |
| 2 | Malpresentation / unstable lie |
| 3 | Multiple pregnancy |
| 4 | Presumed fetal compromise /IUGR / abnormal CTG |
| 5 | Cord prolapse |
| 6 | Chorioamnionitis |
| 7 | Other fetal |
| 8 | Placenta previa actively bleeding |
| 9 | Placenta previa not actively bleeding |
| 10 | Antepartum / intrapartum hemorrhage |
| 11 | Placental abruption |
| 12 | Pre-eclampsia / eclampsia |
| 13 | Maternal medical disease |
| 14 | Failure to progress (induction / in labor) |
| 15 | Previous CS |
| 16 | Uterine rupture |
| 17 | Maternal request |
| 18 | Previous poor obstetric outcome |
| 19 | Previous physically or emotionally traumatic VD |
| 20 | Previous infertility |
| 21 | Other maternal |

**NICE 2004: Evidence based planned CS**

| **Category** | **Recommendation** | **Grading of recommendation*** |
| --- | --- | --- |
| Breech presentation | 1. Women who have an uncomplicated singleton breech pregnancy at 36 weeks gestation should be offered external cephalic version. Exceptions include women in labour and women with a uterine scar or abnormality, fetal compromise, ruptured membranes, vaginal bleeding or medical conditions. 2. Pregnant women with a singleton breech presentation at term, for whom external cephalic version is contraindicated or has been unsuccessful, should be offered CS as it reduces perinatal mortality and neonatal morbidity. | A  A |
| Multiple pregnancy | 1. In otherwise uncomplicated twin pregnancies at term where the presentation of the first twin is cephalic, perinatal morbidity and mortality is increased for the second twin. 2. However, the effect of planned CS in improving outcome for the second twin remains uncertain and therefore CS should not routinely be offered outside a research context. 3. In twin pregnancies where the first twin is not cephalic the effect of CS in improving outcome is uncertain but current practice is to offer a planned CS. 4. Planned CS for uncomplicated twin pregnancy should not be carried out before 38 weeks because this increases the risk of respiratory problems in these babies. | C  GPP  B |
| Preterm birth | 1. Preterm birth is associated with higher neonatal morbidity and mortality. However, the effect of planned CS in improving these outcomes remains uncertain and therefore CS should not routinely be offered outside a research context. | B |
| Small for gestational age | 1. The risk of neonatal morbidity and mortality is higher with ‘small for gestational age’ babies. However, the effect of planned CS in improving this outcome remains uncertain and therefore CS should not routinely be offered outside a research context. | C |
| Placenta praevia | 1. Women with a placenta that partly or completely covers the internal cervical os (grade 3 or 4 placenta praevia) should be offered CS. | D |
| Predicting CS for cephalopelvic disproportion in labour | 1. Pelvimetry is not useful in predicting “failure to progress” in labour and should not be used in decision making about mode of birth. 2. Shoe size, maternal height and estimations of fetal size (ultrasound or clinical examination) do not accurately predict cephalopelvic disproportion and should not be used to predict “failure to progress” during labour. | A  B |
| Mother-to-child transmission of maternal infections | 1. HIV-positive women who are pregnant should be offered a planned CS because it reduces the risk of mother-to-child transmission of HIV. 2. Mother-to-child transmission of hepatitis B can be reduced if the baby receives immunoglobulin and vaccination. In these situations pregnant women with hepatitis B should not be offered a planned CS because there is insufficient evidence that this reduces mother-to-child transmission of hepatitis B virus. 3. Women who are infected with hepatitis C should not be offered planned CS because this does not reduce mother-to-child transmission of the virus. 4. Pregnant women who are co-infected with hepatitis C virus and HIV should be offered a planned CS as this reduces the mother-to-child-transmission of both 5. hepatitis C virus and HIV. 6. Women with primary genital herpes simplex virus (HSV) infection occurring in the third trimester of pregnancy should be offered planned CS because it decreases the 7. risk of neonatal HSV infection. 8. Pregnant women with a recurrence of HSV at birth should be informed that there is uncertainty about the effect of planned CS in reducing the risk of neonatal HSV infection. Therefore, CS should not routinely be offered outside a research context. | A  B  C  C  C  C |
| Maternal request | 1. Maternal request is not on its own an indication for CS and specific reasons for the request should be explored, discussed and recorded. 2. When a woman requests a CS in the absence of an identifiable reason, the overall benefits and risks of CS compared with vaginal birth should be discussed and recorded. 3. When a woman requests a CS because she has a fear of childbirth, she should be offered counselling (such as cognitive behavioural therapy) to help her to address her fears in a supportive manner, because this results in reduced fear of pain in labour and shorter labour. 4. An individual clinician has the right to decline a request for CS in the absence of an identifiable reason. However the woman’s decision should be respected and she should be offered referral for a second opinion. | GPP  GPP  A  GPP |

*GPP = Good practice point based on the view of the Guideline Development Group

**Gregory 1994: Indications for repeat CS**

| **Category** | **Subcategory** |
| --- | --- |
| **Breech** | Supersedes all other codes |
| **Dystocia** | Arrest disorders  Failed vacuum or forceps  Failed induction of labour  Fail trial of labour |
| **Fetal distress** | (before or during labour)  Cord prolapse |
| **Other** | Placenta previa  Malpresentation (excludes breech)  Fetal growth retardation  Herpes simplex virus  Macrosomia  Diabetes mellitus  Hypertensive disorders  Chorioamnionitis  Prior classical cesarean  Prior uterine rupture  Prior myomectomy involving endometrial cavity  Other maternal conditions |
| **Elective repeat cesarean section** | **(Excludes all above)**  Refused trial of labour  Not otherwise specified (presumed elective) |

**Nico 1990: Indications: Programmed or not, with dystocia or not**

| **Category** | **Subcategory** | **Definition** |
| --- | --- | --- |
| Type A: programmed CS |  |  |
| Type B: Not programmed CS but not due to failure to progress |  |  |
| Type C: Failure to progress or dystocia | 1. Prolonged latent phase | Regular contractions at ≥2/10 min and dilation in 2-3 or 4 cm for more than 4h. |
|  | 2. Prolonged active phase (protraction) | Dilation ≤1cm/h with ≥5cm dilation for any parity |
|  | 3. Arrest of dilation (secondary) | Arrest of dilation for ≥2h at 5-7cm inclusive |
|  | 4. Prolonged deceleration phase | Failure to progress at 8-9cm; not complete dilation and CS before complete dilation |
|  | 5. No desent | CS with complete dilation without desent (flouting or insinuated presentation |
|  | 6. Arrested decsent | CS with complete dilation and arrest of decent. Presentation on 2 or 3 plane |

**Stanton 2008: Absolute maternal vs non absolute indications for CS**

| **Category** | **Subcategory** |
| --- | --- |
| Absolute maternal (hierarchical)  **(A)** | 1. Obstructed labour    1. Severe deformed pelvis    2. Fail trial of labour 2. Major antepartum haemorrhage    1. Grade 3 and 4 placenta praevia 3. Malpresentation    1. transverse    2. oblique    3. brow 4. Uterine rupture |
| Non absolute indications (non hierarchical)  **(NA)** | 1. Failure to progress in labour    1. Prolonged labour    2. Failed induction 2. Previous CS 3. Genitourinary fistula or third-degree tear repair 4. Antepartum haemorrhage excluding those for absolute indication and including abruption 5. Maternal medical diseases 6. Severe pre-eclampsia or eclampsia 7. Psychosocial indications 8. Fetal compromise including fetal distress, cord prolapse, severe IUGR 9. Breech presentation |

**Unmet Needs Network Classification 2000**

| **Category (Absolute Maternal Indications)** | **Subcategory** |
| --- | --- |
| Malpresentations and malpositions | Breech  Shoulder  Face and brow  POP and DTA* |
| Antepartum Haemorrhage | Placenta previa  Abruptio placentae  Unspecified APH* |
| Maternal diseases | Severe PET/eclampsia*  Diabetes  Other diseases |
| Fetal reasons | Cord prolapse  Feto-pelvic disproportion  Fetal distress |

* No definitions given by authors on meaning of : POP, DTA, APH or PET

**Cisse 1998: Senegalese 3 groups of indications for CS**

| **Category** | **Definition** | **Subcategory** |
| --- | --- | --- |
| 1. Obligatory | Vaginal delivery is not possible. The absence of an intervention will lead to maternal or fetal death or another serious outcome | a. Cephalo-pelvic disproportion  b. Placenta previa  c. Dystocia |
| 2. Prudent | Vaginal delivery is theoretically possible but CS improves the mother's or baby's prognosis. | a. Scarred uterus  b. Breech  c. Fetal distress  d. Precious fetus (older primipara in high fertility context) |
| 3. Necessary | CS performed at last resource, resulting from poor management of labour or delayed referral | a. Hypertension  b. Dystocia dinamic |

**Kushtagi 2008: Documentation of indication for delivery and for CS**

| **Category** | **Subcategory** | **Note** |
| --- | --- | --- |
| 1. Indication for termination of pregnancy |  |  |
| 2. Indication for cesarean delivery | (a) immediate compelling indication |  |
|  | (b) Associated indications | - If present - to be listed in order of importance; - only two to be listed |
|  | (c) High risk factors | - if present - only two to be listed |
|  | (d) Perioperative/neonatal | (i) finding supporting  (ii) indication (if present) |

**Classifications based on degree of urgency**

**Van Dillen 2009 (a): Lucas classification with additional interpretation**

| **Category** | **Level of urgency** | **Additional interpretation** |
| --- | --- | --- |
| Immediate threat to the life of mother of fetus | 1 | CS is performed for acute life-threatening events. There is an emergency situation; CS should be performed as soon as possible to save the life of mother or fetus. |
| Maternal or fetal compromise but not immediately life threatening | 2 | Delivery of the fetus is urgent, because maternal or fetal compromise is present and it is demonstrated at this moment. CS is needed to prevent deterioration of either maternal or fetal condition. |
| The mother needed early delivery but there was no maternal or fetal compromise | 3 | No maternal or fetal compromise is present at this moment, but compromise may be expected if spontaneous delivery is awaited. |
| Delivery was timed to suit the mother or the staff | 4 | Compromise is not expected if CS is not performed. There is no strict medical indication. |

**Nicopoullos 2003 (a): Priority of delivery for CS**

| **Category** | **Level of urgency** |
| --- | --- |
| **Crash** | 10-20 minutes |
| **Urgent** | Within 30 minutes |
| **Emergency** | Within 2 hours |
| **Elective** | No time required |

**Lucas 2000: Urgency of CS Classification based on clinical definition**

| **Category** | **Level of urgency** | **Level of urgency** |
| --- | --- | --- |
| Immediate threat to the life of mother of fetus | Emergency | 1 |
| Maternal or fetal compromise but not immediately life threatening | Urgent | 2 |
| The mother needed early delivery but there was no maternal or fetal compromise | Scheduled | 3 |
| Delivery was timed to suit the women and maternity team | Elective | 4 |

**Van Dillen 2009 (b): Traditional Binary System for Degree of Urgency of CS**

| **Category** | **Definition** |
| --- | --- |
| Primary | if vaginal delivery was not intended—even if the woman presents in labor |
| Secondary | if vaginal delivery was attempted. |

**Huissoud 2009: Color codes for urgency of CD**

| **Category** | **Level of urgency** | **Definition/Inclusion** |
| --- | --- | --- |
| **Green code** | Non-urgent CS  Decision-to-delivery interval ≤1h | There is a need for early delivery but no compromise or threatening situation is seen at short-term   - Failure to progress, dilation or desent - Dystoc presentations - Prophilactic CS |
| **Orange code** | Urgent CS  Decision-to-delivery interval ≤30 min | There is a need for CS because there is a short-term threat for the prognosis of the mother or the fetus |
| **Red code** | As soon as possible urgent CS  Decision-to-delivery interval ≤15 min | This is an emergency situation; CS should be performed as soon as possible to save the life of mother or fetus. CS is performed for acute life-threatening events. There is life-threatening situation. |

**Classifications based on women's characteristics**

**Robson 2001: 10 group system**

| **Group** | **Obstetric population*** |
| --- | --- |
| 1 | Nulliparous with single cephalic pregnancy, ≥37 wks gestation in spontaneous labour |
| 2 | Nulliparous with single cephalic pregnancy, ≥37 wks gestation who either had labour induced or were delivered by CS before labour |
| 3 | Multiparous without a previous uterine scar, with single cephalic pregnancy, ≥37 wks gestation in spontaneous labour |
| 4 | Multiparous without a previous uterine scar, with single cephalic pregnancy, ≥37 wks gestation who either had labour induced or were delivered by CS before labour |
| 5 | All multiparous with at least one previous uterine scar, with single cephalic pregnancy, ≥37 wks gestation |
| 6 | All nulliparous women with a single breech pregnancy |
| 7 | All multiparous women with a single breech pregnancy including women with previous uterine scars |
| 8 | All women with multiple pregnancies including women with previous uterine scars |
| 9 | All women with a single pregnancy with a transverse or oblique lie, including women with previous uterine scars |
| 10 | All women with a single cephalic pregnancy ≤36 wks gestation, including women with previous scars |

*CS rates and size of the obstetric population are calculated in each group

**Denk 2006: 8 group system**

|  | **Category** | **Subcategories** |
| --- | --- | --- |
| Primary CS | Class 1: Standard* nullipara  Class 2: Standard multipara  Class 3: Malpresentation^#^ nullipara  Class 4: Malpresentation multipara  Class 5: All multiple gestation  Class 6: Singleton preterm | For each category:   1. No trial of labour 2. Induced labour 3. Spontaneous |
| Repeated CS | Class 7: Standard with prior CS  Class 8: All other with prior CS |  |

* Standard = Term, singleton, cephalic

^#^ Malpresentation = Breech, oblique and transverse

**Cleary 1996: Standard primipara**

| **Category** | **Definition** |
| --- | --- |
| Standard primipara | White, aged 20-34 years, more than 155 cm tall, delivering a singleton cephalic fetus of more than 37 completed weeks of gestation in the unit at which she originally booked, **excluding** cases subject to medical complications of pregnancy:  Chronic systemic diseases that predate pregnancy:   - Diabetes - Hypertension - Epilepsy - cardiac disease - renal disease   as well as certain medical complications arising during the pregnancy:   - diabetes - severe anaemia (Hb<10g) - APH - Hypertension (diastolic >96) |

**Lieberman 1998: Case Mix Model for adjusting CD rates**

This author presents the classification as a matrix. 18 categories have been considered in this classification

| **Obstetric condition category***^#^ | **Nulliparas**  **(A)** | **Multiparas no previous CS (B)** | **Multiparas with at least one previous CS**  **(C)** |
| --- | --- | --- | --- |
| 1. Multiple pregnancy |  |  |  |
| 2. Breech/transverse lie presentation |  |  |  |
| 3. Preterm birth (<36 weeks) |  |  |  |
| 4. No trial of labour permitted for medical reason (women who had a pre-existing or emergent medical indication or CS known at the time of admission)   1. Active genital herpes 2. Placenta previa 3. History of myomectomy 4. Macrosomia 5. Non-reassuring fetal status 6. Other less common medical conditions 7. ≥2 previous CS 8. Previous classic cesarean scar |  |  |  |
| 5.≥36 weeks permitted to labour with presence of medical risks and complications at the time of admission   1. Hypertension 2. Diabetes 3. Abruption placenta 4. Oligohydramnios 5. Polyhydramnios 6. Fetal growth restriction 7. Fetal hydrops 8. Non-reassuring fetal condition 9. Fever ≥100.4 F 10. Infants weighing ≥4500g |  |  |  |
| 6. ≥36 weeks permitted to labour without presence of medical risks or complications at the time of admission |  |  |  |

* Classification criteria based on conditions present at admission since they adjusted for conditions associated with an increased risk of CS that were largely independent of management decisions but rather reflected the characteristics of the population.

^#^ Women were classified into the first group to which she belonged and therefore groups are mutually exclusive.

**Other types of classifications**

**RCOG 2001 (b): Organizational and Staffing factors (Sentinel Audit)**

| **Organizational factor** | **Category** |
| --- | --- |
| Size of maternity unit | <1000  1000-1999  2000-2999  3000-3999  4000-5999  >6000 |
| Presence of neonatal intensive care unit or special care baby unit | Yes or No  (and analysis by number of cots) |
| Being a tertiary referral center | Yes or No |
| Affiliation with a medical school | Yes or No |
| Availability of 24-hour anaesthetist | Yes or No |

**RCOG 2001 (c): Potentially complicated cesarean sections**

| **Category** |
| --- |
| Placenta praevia |
| Placental abruption |
| At full cervical dilatation |
| In obese women |
| For premature delivery <32 weeks |
| For multiple pregnancy |
| In women who have had multiple CS |

**Nicopoullos 2003 (b): Documentation of obstetric care in CS**

| **Category/Item to document** | **%** |
| --- | --- |
| **Recording of indication and clinician details**  Indication  Name of surgeon  Grade of surgeon  Name of assistant  Name of anaesthetist  Type of anaesthetic |  |
| **Recording of indication and clinician details**  Skin incision time  Skin incision type  Surgical findings  Uterine incision type  Engagement of presenting part  Fetal delivery  Placenta delivery  Uterine cavity check  Presence of paediatrician  Adnexal check  Estimate of blood loss  Post-op care plan |  |

**ICD-10 1992: Classification for CS**

| **Category** | **Description** |
| --- | --- |
| **O82.0** | Single delivery by elective CS |
| **O82.1** | Single delivery by emergency CS |
| **O82.2** | Single delivery by cesarean hysterectomy |
| **O82.8** | Other single delivery by CS |
| **O82.9** | Single delivery by CS, unspecified |
| **O84.2** | Multiple delivery, all by CS |
| **O84.8** | Other multiple delivery by combination of methods |

**WHO 2004: Global survey classification for CS**

(only for CS outcome)

| **Category** |
| --- |
| **Elective CS, no labour** |
| **Emergency CS, no labour** |
| **Intrapartum CS** |

**Guidotti (Unpublished): Safety of CS in resource poor settings**

| **Element / Category** | **Subcategory** |
| --- | --- |
| Necessity for a C-section | 1. CS-A a condition that has the highest probability of a better outcome that a vaginal delivery. 2. CS-B a condition that has a high probability of a better outcome than vaginal delivery. 3. CS-C a condition that has an equal probability of the same outcome than a vaginal delivery. |
| Condition of woman | 1. CW-A health status of mother is uncompromised 2. CW-B health status of mother moderately compromised 3. CW-C health status of mother severely compromised |
| Condition of the fetus | 1. CF-A health status of fetus is uncompromised 2. CF-B health status of fetus is moderately compromised 3. CF-C health status of fetus is severely compromised |
| Surgical Team | - - 1. Surgeon        - 1. SU-A Board qualified, active          2. SU-B Resident 3-4 years, active          3. SU-C Resident (newly trained inexperienced)     2. Anesthesiologist        - 1. AN-A Board qualified, active          2. AN-B Resident          3. AN-C Resident (newly trained inexperienced) |
| Surgical Procedure | - - 1. SP-A Cesarean performed according to best practices/evidence based methods     2. SP-B Cesarean performed sub-standard (but approximates best practices)     3. SP-C Cesarean performed sub-standard does not approximate best practice) |
| Anaesthesia Procedure | - - 1. AP-A Anaesthesia performed according to best practice/evidence based     2. AP-B Anaesthesia performed sub-standard (approximates best practice)     3. AP-C Anaesthesia performed sub-standard (does not approximate best practice) |
| Surgical Instruments | 1. SI-A Complete set in optimal condition 2. SI-B Incomplete set missing items moderately compromised 3. SI-C Incomplete set missing items severely compromised |
| Anaesthesia equipment | 1. AE-A Complete essential available 2. AE-B Incomplete moderately compromised 3. AE-C Incomplete severely compromised |
| OT condition | - - - 1. OT-A Excellent all equipment necessary for safe surgery       2. OT-B Fair not all equipment present, malfunctioning       3. OT-C Poor severely missing items, malfunctioning |
| Drugs | - - - 1. DR-A Excellent: all necessary drugs for pre-op, op (incl anaesthesia) & po are available, within expiration dates, sufficient amounts.       2. DR-B Fair, not all necessary drugs (see above) available, etc….       3. DR-C Poor, severely lacking necessary drugs (see above)…. |
| Post-operative care for woman | - - - 1. PW-A Excellent: Post operative care given according to best practice guidelines       2. PW-B Fair: Post operative care sub-standard (approx to best practice guidelines       3. PW-C Poor: post operative care sub-standard (does not approx to best practice guidelines. |
| Post-operative care for neonate | - - - 1. PN-A Excellent: Post operative care given according to best practice guidelines  1. PN-B Fair: Post operative care sub-standard (approx to best practice guidelines 2. PN-C Poor: post operative care sub-standard (does not approx to best practice guidelines. |
